# Respecting Partial Privacy of Unstructured Data via Spectrum-Based Encoder

**DOI:** 10.3390/s24031015

**Published:** 2024-02-04

**Authors:** Qingcai Luo, Hui Li

**Affiliations:** 1 School of Cyber Engineering, Xidian University, Xi’an 710126, China; 2 School of Computer Science and Technology, Xidian University, Xi’an 710071, China; hli@xidian.edu.cn

**Keywords:** spectrum-based encoder, latent code, machine learning, privacy preserving

## Abstract

Since the popularity of Machine Learning as a Service (MLaaS) has been increasing significantly, users are facing the risk of exposing sensitive information that is not task-related. The reason is that the data uploaded by users may include some information that is not useful for inference but can lead to privacy leakage. One straightforward approach to mitigate this issue is to filter out task-independent information to protect user privacy. However, this method is feasible for structured data with naturally independent entries, but it is challenging for unstructured data. Therefore, we propose a novel framework, which employs a spectrum-based encoder to transform unstructured data into the latent space and a task-specific model to identify the essential information for the target task. Our system has been comprehensively evaluated on three benchmark visual datasets and compared to previous works. The results demonstrate that our framework offers superior protection for task-independent information and maintains the usefulness of task-related information.

## 1. Introduction

Machine learning has demonstrated impressive performance in several areas, such as natural language processing [1] and computer vision [2]. However, training an effective machine learning model requires proper model design, massive computing resources, and large datasets that may be beyond the reach of many individuals. In addition, deploying and running the model requires significant storage and computing resources that are also unfriendly to edge devices such as smartphones or sensors [3]. One promising approach is Machine Learning as a Service (MLaaS) [4], which supports the outsourcing of prediction. Well-trained models can be deployed by vendors in the cloud. This is attractive because it offloads the user’s local computing and storage requirements and eliminates the cost of training new models. However, the outsourced data consist of not only task-related information, but also task-independent information [5], which does not significantly affect the inference results, but exposes users to unwanted risks of misuse or theft. Recently, China’s Personal Information Protection Law has prompted information processors to prevent unauthorized access to personal information. Therefore, it is of paramount importance to protect unauthorized information while ensuring the usefulness of the data.

Previous works addressing privacy concerns have been devoted to balancing the trade-offs between privacy and utility. An obvious and widely adopted solution is to extract task-oriented features and upload them to servers instead of raw data, such as Google Now [6] and Google Cloud [7]. Although the mere transmission of features avoids direct disclosure of raw data, recent developments in model inversion attacks show that adversaries can use intermediate features to reconstruct the input and infer privacy attributes [8,9,10]. Ossia et al. [11] apply dimensionality reduction and noise injection to defend against adversaries before uploading features to the servers, but the cost is a non-negligible loss in utility. Inspired by Generative Adversarial Networks (GANs) [12], PAN [13], DeepObfuscator [3], and TIPRDC [14] propose to obtain an encoder through adversarial training to extract partial privacy-preserving features that keep a subset of the attributes available while specifying the attributes anonymously. However, these schemes artificially simulate proxy adversaries during the training phase, leading to dangers from potential attack models. This suspicion is also supported by the results of the potential adversary detection experiments in Section 4.2.

**Figure 1:**
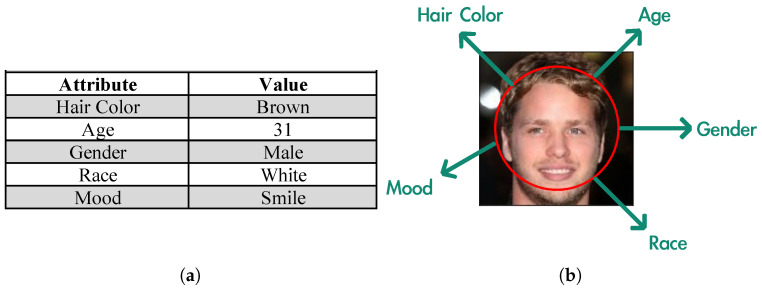
(**a**) Structured data and (**b**) unstructured data.

Therefore, we propose a partial privacy-preserving framework to preserve data utility while protecting task-independent attributes. An intuitive phenomenon is that not all data information is useful for inference. Some of the recent literature shows that the task model pays more attention to a part of regions [15,16,17,18], which becomes evidence that the data can be regarded as composed of task-related and task-independent information. Inspired by these works, our framework focuses on selecting the information relevant to the target task. This is feasible for structured data, but difficult for unstructured data. Taking Figure 1 as an example, users can flexibly select the attributes necessary for the task in Table (a) due to the naturally independent entries, while it is impractical for image (b) because different attributes are entangled and expressed in the same region. An intuitive approach is to express unstructured data in a structured form. However, naturally occurring data are often accompanied by redundant information, which hinders structured expression. Therefore, we introduce Fourier transform as a pre-processing method to reduce data redundancy, and propose the spectrum-based encoder to disentangle the unstructured data into a latent space [19]. We then propose a universally interpretable model, called an indicator, which marks the information necessary for the target task in the latent representation.

**Figure 2:**
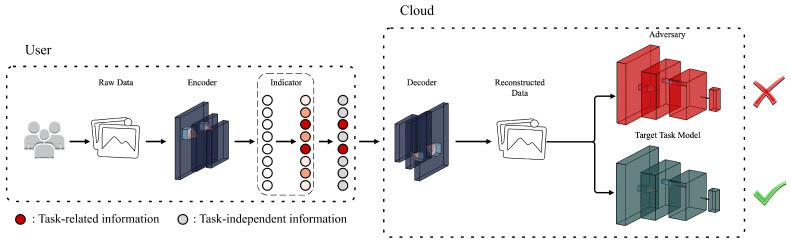
The VAE-based encoder maps the raw data to the latent space, and the proposed indicator points out the relevance of the latent code to the target task and removes irrelevant elements. The subsequent decoder reconstructs the data from the filtered code, with the target attributes being preserved while the remaining attributes are obfuscated.

As shown in Figure 2, our framework consists of three parts: a spectrum encoder *E*, an indicator *I*, and a decoder *D*. The encoder *E* is intended to be used on the user side to extract the disentangled representation from unstructured data. Indicator *I* is also used on the user side, recommending task-related information by marking representation dimensions. The marked dimensions indicate the information required by the target task model, and the corresponding anonymized transform is designed. Specifically, the values of the marked dimensions are retained, while the values of the ignored dimensions are discarded and reassigned as default values. The decoder *D* runs on the server to reconstruct the data based on the transformed representation uploaded by the users. The classifier (green) is considered the target task model, and the reconstruction data will strive to maintain its usefulness. At the same time, the reconstruction data are expected to prevent adversaries (red) from inferring unauthorized attributes.

Discarding task-independent information according to Indicator’s recommendations has four advantages. First, interpretable indicators provide interpretability for anonymized transformation. Second, target-task-driven attribute retention avoids unconscious utility loss and sensitive information leakage. Third, disentangled representation-based information selection provides an explicit and controllable balance for privacy–utility trade-offs. Finally, this allows our framework to withstand potential attack models. Furthermore, Indicator and encoder–decoder pairs of our framework are trained separately in two phases. Compared to existing end-to-end adversarial training methods, our framework can adapt to the changes in the target tasks and adjust the retained attribute information more flexibly.

In summary, our key contributions are as follows:

- We introduce a novel interpretable model called Indicator, which can effectively indicate the critical information required for a specific target task within unstructured data.
- We present a partial privacy-preserving framework that utilizes the designed Indicator to restrict the access of undesired task-independent attacks while preserving the utility of target tasks.
- We fully implement our framework and demonstrate its wide applicability by performing experiments on several standard datasets. The evaluation results show that our framework can achieve sweet trade-offs between privacy and utility, and is resistant to potential attackers.

The rest of this paper is organized as follows. Section 2 introduces the preliminaries and reviews the related work. Section 3 describes the framework overview and the details of core modules. Section 4 reports the evaluation results. Section 5 concludes and discusses this paper.

## 2. Preliminaries and Related Work

In this section, we first introduce the work involved in this article. Then, we briefly review the most relevant work on privacy.

### 2.1. Disentangled Representation Learning

**Figure 3:**
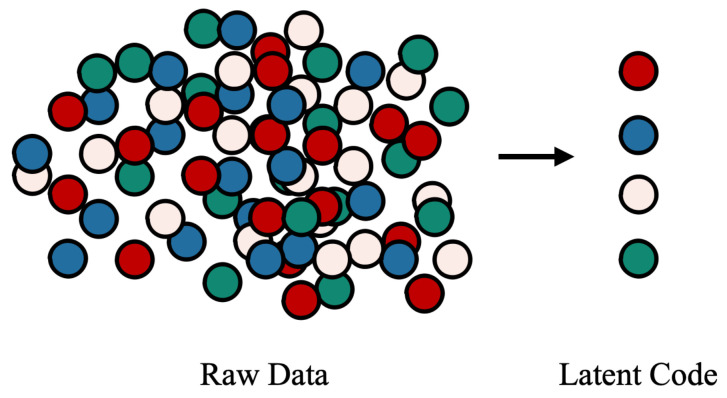
Different colours represent different attributes in the unstructured data, and the balls represent the factors that affect the attributes.

In general, disentangled representation learning aims to isolate different attributes into non-overlapping sub-dimensions in the latent space. As shown in Figure 3, different colours represent different attributes in the raw data, and the ball represents the factor containing attribute information. In the raw data, these factors are messy and entangled, and it is difficult to filter all the factors corresponding to a certain attribute in a common way. At the same time, the latent code obtained by the disentangled representation learning can express attributes regularly and independently. In other words, different attributes in the raw data can be determined by the different representation sub-dimensions in the latent space.

Existing works about disentangled representation can be roughly divided into three categories: (1) based on Variational Autoencoders (VAE) [19,20,21], (2) based on GAN [22] and (3) based on the flow model [23]. Among them, the VAE-based model is attractive due to its lower cost and stability in the training phase.

VAE is an unsupervised generative network based on variational bayes inference, consisting of an encoder and a decoder. Given a sample x, VAE determines a distribution z in the latent space as the encoding result. The optimization objective of VAE consists of two parts. The first part is to maximize the Evidence Lower Bound (ELBO) so that the variational distribution is close to the isotropic Gaussian prior p(z), and the second part is to minimize the pixel-level metrics of the generated data and the original data:

(1) $$\begin{array}{c} L_{VAE}=-E_{q(z|x)}[log\left(p\left(x|z\right)\right]+D_{KL}\left(q\left(z|x\right)||p\left(z\right)\right) \end{array}$$

β-VAE [19] modified the objective function as:

(2) $$\begin{array}{c} L_{\beta VAE}=-E_{q(z|x)}[log\left(p\left(x|z\right)\right]+\beta D_{KL}\left(q\left(z|x\right)||p\left(z\right)\right) \end{array}$$

Compared to the original VAE, the hyperparameter β>1 encourages the variational distribution to be closer to the Gaussian prior, thereby producing a disentangled latent code. Kim et al. [20] and Chen et al. [21] believed that the total correlation term obtained by decomposing the KL divergence plays a crucial role and proposed Factor-VAE and β-TCVAE, respectively.

### 2.2. Data Privacy Protection

Several methods have been proposed to protect privacy. k-anonymity [24], l-diversity [25], and t-closeness [26] have been proposed as desensitization criteria. However, these methods are only designed for structured data and are difficult to scale to unstructured data. Differential privacy [27,28,29] and random noise injection [30,31] are common methods that are widely used to protect sensitive information in structured and unstructured data. Although security guarantees are provided, these methods often significantly reduce the usefulness of the data. Homomorphic encryption (HE) [32,33] and Secure Multi-Party Computation (MPC) [34,35,36] support the manipulation of encrypted data, but the computation of non-linear functions is always accompanied by unrealistic computational and communication complexity, leading to much lower efficiency than plaintext inference. iPrivacy [37] focuses on visual tasks by constructing a multi-task learning model to detect and blur objects that may leak sensitive information in the image. The types of these objects are preset. RAE [38] follows the same idea but is time-series-oriented. This scheme proposes to replace the features of each section corresponding to sensitive inferences with the values corresponding to non-sensitive inferences. Using GAN, RAE provides the security guarantee that it is almost impossible to detect the nature of sensitive inferences.

### 2.3. Representation Privacy–Utility Trade-Offs

Aloufi et al. [39] focused on the disentanglement of voice for the Voice User Interfaces (VUIs). VQ-VAE [40] was introduced to construct independent representations of emotion, identity, and semantics, while WaveRNN [41] was employed to reconstruct voice information. Gong et al. [42] are concerned about attributes preserving face de-identification and propose $R^{2}VAEs$ to obfuscate identity-related information so as to achieve a balance between facial privacy protection and data utilities. Wu et al. [43] jointly proposed a securely recoverable visual information transformation and steganography PECAM based on deep learning. They used this technology to design a more general VSA privacy enhancement architecture and system implementation. PECAM can effectively transform the original data to other domains to hide sensitive information. At the same time, authorized users can inversely transform and restore the original data to complete detailed investigations. This secure reversible transformation relies on a security-enhanced generative adversarial network. Also, it introduces a key mechanism to ensure that attackers cannot restore the data protected by PECAM. The adversary and the defender are given the conflicting utility–privacy optimization goal, and the game between them is simulated. AttriGuard [44] proposed a two-phase practical framework to resist private attribute inference attacks. In phase I, existing evasion attacks in adversarial learning are adopted to find the minimum noise for each attribute value. In phase II, the attribute values are sampled with a certain probability distribution, and the minimum noise found in phase I is added to the dataset. Therefore, finding the probability distribution is formulated as a constrained convex optimization problem. Liu et al. proposed PAN to protect the privacy of a specific attribute while maintaining the data utility for a certain task. The representation obtained by PAN will remain anonymous, and the adversaries cannot launch reconstruction attacks or privacy attributes inference attacks. Wu et al. [45] designed an adversarial training framework to obtain the degradation transform of video inputs to resist privacy attribute attacks. Considering the diversity of attack models, and that it is impossible to enumerate all adversary models to enhance the features privacy, Budget Model Restarting and Budget Model Ensemble are enabled to enrich potential adversaries. TIPRDC is a task-independent privacy-respecting data crowdsourcing framework but following the same idea. Unlike the above works, the data utility maintained by TIPRDC does not limit to specific tasks but is effective for arbitrary tasks by maximizing mutual information. In a sense, our work is diametrically opposed to the idea of TIPRDC: TIPRDC struggles to **retain** all information in the data, except for privacy attributes, while our framework is expected to **remove** all information, except for the target task required.

## 3. Design of Framework

In this section, we introduce the VAE-based disentanglement method and propose the model called Indicator for filtering the factors related to the target task.

### 3.1. Overview

Because models do not need all the information in the uploaded data to make credible inferences, users tend to share only task-relevant details in a controlled manner. This is practical for structured data with naturally independent attribute records but is difficult for unstructured data. Thus, our framework is proposed to sift task-related information from the unstructured data while confusing task-independent information. Figure 2 shows that our framework addresses this problem in three stages. In the first stage, the encoder in the VAE family model is used to obtain the disentangled representation, from which different attribute information can be independently selected. Although the disentangled representation is similar in form to the structured data, users are still confused about which dimensions are necessary due to the lack of semantic interpretation. Therefore, in the second step, we propose a model called Indicator that provides suggestions for explicit user control over the information. In the representation, the dimensions marked by Indicator are frozen, while the values of the remaining dimensions are discarded and refilled. In the third stage, the transformed representation is fed to the decoder that corresponds to the encoder in the first stage for data reconstruction. The task-related information in the reconstructed data is preserved, while the task-independent information is unreliable.

### 3.2. Unstructured Data Disentanglement

**Figure 4:**
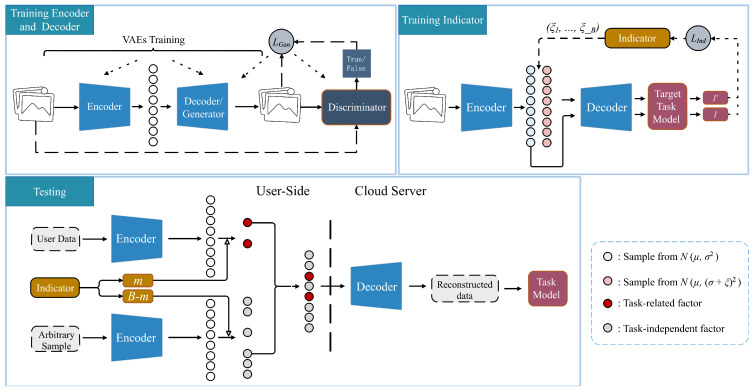
The workflow of our framework. The top line is the training stage, including the training of the encoder–decoder pair and Indicator. The bottom line is the test stage. An indicator is introduced to recommend the indexes of the representation dimensions that need to be retained. At the same time, an arbitrary sample is used as a carrier to supplement the remaining dimensions.

The information of different attributes in unstructured data is often intertwined and almost impractical to select independently. By disentangling different attributes, it is possible to preserve the task-related factors of unstructured data while obfuscating the task-independent factors. As shown in Figure 4, we employ the VAE family models (β-VAE, Factor-VAE, and β-TCVAE) in the training phase to obtain an encoder–decoder pair. The encoder is used to extract the disentangled representation, and the decoder is used to reconstruct the data. However, the common problem is that the data generated by VAEs is always ambiguous. One view is that the pixel-wise reconstruction error metric causes the generated data to be too smooth [46]. In contrast, the main idea of GAN is to provide a game between the generator and the discriminator. During this game, the discriminator judges the original data as true and the generated data as false at each iteration. Meanwhile, the generator tries to fool the discriminator into judging the generated data as true in the same iteration. Therefore, the decoder can be considered as the generator and a discriminator is introduced to improve the quality of the generated data. To avoid affecting the disentanglement of the representation, in each iteration, the training of the GAN is carried out after the training of the VAEs, which means that the encoder and the discriminator are not end-to-end. Formally, the loss function can be defined as:

(3) $$\begin{array}{c} L_{VAEs}\left(\theta_{End},\theta_{Dec}\right)=L_{VAEs} \end{array}$$

(4) $$\begin{array}{c} L_{GAN}\left(\theta_{Dec}\right)=E_{x}\left[log\left(Dis\left(x\right)\right)+log\left(1-Dis\left(x^{\prime }\right)\right)\right] \end{array}$$

(5) $$\begin{array}{c} L_{GAN}\left(\theta_{Dis}\right)=-E_{x}[log\left(log\left(Dis\left(x^{\prime }\right)\right)\right] \end{array}$$

where $L_{VAEs}$ represents different loss functions in the VAE family and $\theta_{*}$ indicate the parameters to be updated.

In the testing phase, the encoder is deployed on the user side while the corresponding decoder runs on the cloud server.

### 3.3. Representation Oriented Indicator

After the encoder and decoder training, the encoder can standard express different attributes in the latent space. Such a disentangled representation allows us to obfuscate the task-independent factors without changing task-related factors. However, data contain many factors, and it is impractical to enumerate all task-independent attributes. In addition, whether a factor is related to the task depends on the specific task model. Different classifiers may focus on different associated attributes for the same classification task. For example, one classifier will concentrate on hair when judging the gender of a face image, while another classifier may focus on beards. The tendency of the classifier depends on the training set and model structure, which is uncontrollable for the user. If the factors to be obfuscated are rashly determined based on human perception, it will inevitably affect the effectiveness of the primary task. For this consideration, the task-adaptive Indicator is proposed to mark the attributes that the specific task model focuses on.

Different dimensions in the disentangled representation are considered disjoint, and a set of sub-dimensions can only express a particular data attribute. Meanwhile, the task model does not view all the information to make credible inferences but pays more attention to specific attributes. This is equivalent to that only one set of sub-dimensions in the disentangled representation contributes to the task model inference while discarding the values of the remaining dimensions has almost no effect. Following the idea, the proposed Indicator is designed to search this set of sub-dimensions. Indicator is expected to have both fidelity and interpretability. Fidelity means that Indicator can accurately mark the representation dimensions necessary for the task model. The interpretability signifies that the decision-making process is consistent with the human perspective.

**Figure 5:**
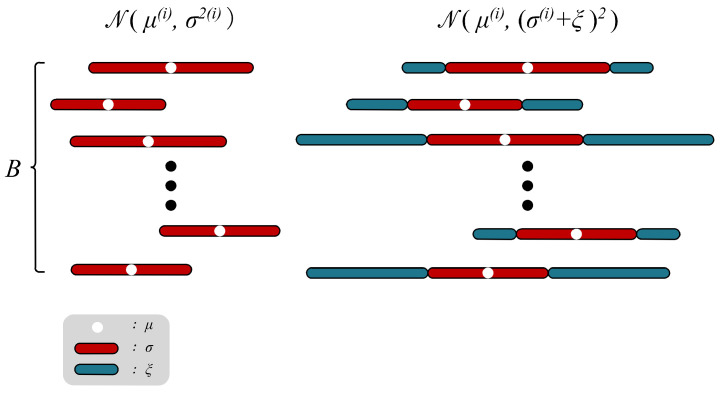
Illustration of how the indicator works. Indicator searches for the maximum allowable oscillation range that remains utility for the task model in the *B* representation dimensions.

Figure 5 reviews the paradigm of the VAE family. Each original datapoint $x^{\left(i\right)}$ is encoded into a multivariate gaussian distribution $N(\mu^{\left(i\right)},{\left(\sigma^{\left(i\right)}\right)}^{2})$, and the decoding results ${x^{\prime }}^{\left(i\right)}$ of all samples in $N(\mu^{\left(i\right)},{\left(\sigma^{\left(i\right)}\right)}^{2})$ are similar to the original data $x^{\left(i\right)}$. Given an original datapoint $x^{\left(i\right)}$, its disentangled representation $z^{\left(i\right)}$ can be represented by $z^{\left(i\right)}∼N\left(\mu^{\left(i\right)},{\left(\sigma^{\left(i\right)}\right)}^{2}\right)$, and $z^{\left(i\right)}\in R^{B}$. For the inference of a certain task model, there are *m* necessary dimensions in $z^{\left(i\right)}$, whose value fluctuation will significantly affect the result of the inference, while the change in the remaining B−m dimensions can hardly have impacts. This demonstrates that under the premise of not affecting the inference confidence, the larger variance is not tolerated by the *m* dimensions, but is acceptable for the B−m dimensions. Therefore, the ultimate goal of Indicator can be expressed as finding a variance bias ξ as large as possible and encoding the data $x^{\left(i\right)}$ into the new distribution $N(\mu^{\left(i\right)},{\left(\sigma^{\left(i\right)}+\xi \right)}^{2})$, as shown in Figure 5. Among them, the decoding result ${\tilde{x}}^{\prime (i)}$ of the sample on $N(\mu^{\left(i\right)},{\left(\sigma^{\left(i\right)}+\xi \right)}^{2})$ and the decoding result ${x^{\prime }}^{\left(i\right)}$ of the sample on $N(\mu^{\left(i\right)},{\left(\sigma^{\left(i\right)}\right)}^{2})$ show the same confidence in the task model. In general, the optimization goal of ξ can be formulated as:

(6) $$\begin{array}{cc} minL(\xi )=min & E_{p(x)}\left[E_{q\left(z|x\right),\;\tilde{q}\left(\tilde{z}|x\right)}\left[L2\left(TM\left(Dec\left(z\right)\right),TM\left(Dec\left(\tilde{z}\right)\right)\right)\right]\right]-\lambda \sum_{i=1}^{B}\xi_{i},\\ q(z|x)=N(\mu , & \sigma^{2}),\;\tilde{q}\left(\tilde{z}|x\right)=N\left(\mu ,{\left(\sigma +\xi \right)}^{2}\right) \end{array}$$

where TM represents the target task model.

After training, the representation dimensions corresponding to the smaller $\xi_{i}$ cannot support the larger sampling ranges while maintaining effectiveness for the task model, which means that the task model will pay more attention to these dimensions. Conversely, the dimensions corresponding to a larger $\xi_{i}$ contribute less to the task model inference. To determine ξ, an intuitive method is to set ξ as trainable parameters. However, there are two problems with this method. First, since Indicator aims to explore the tolerance of different dimensions to the larger variance, $\xi_{i}$ is expected to be non-negative. Also, too large a variance σ+ξ, which leads to meaningless sampling, can cause training to collapse. Therefore, $\xi_{i}$ should be restricted to the interval [0,δ). Second, ξ is the variable in the distribution $N(\mu ,{\left(\sigma +\xi \right)}^{2})$ and the sampling process is not differentiable.

For the first problem, we design a function $\xi_{i}=f\left(\beta_{i}\right),\;\beta_{i}\in R,\;\xi_{i}\in \left[0,\delta \right)$ to eliminate the constraint on ξ, where β is Indicator parameters. Considering the $\lambda \sum_{i=1}^{B}\xi_{i}$ term in Equation (6), f() should also satisfy monotonicity. If $\xi_{i}$ can take the minimum value of 0 when $\beta_{i}=0$, the training of Indicator will benefit from the sparse parameters. Formally, f() can be defined as:

(7) $$\begin{array}{c} \xi_{i}=f\left(\beta_{i}\right)=\delta \cdot \left(\frac{1-e^{-\beta_{i}^{2}}}{e^{-\beta_{i}^{2}}+1}\right) \end{array}$$

Among them, f() is monotonically increasing in [0,+∞), monotonically decreasing in (−∞,0], and the minimum value is 0 at $\beta_{i}=0$. In order to solve the second problem, we borrow the reparameterization trick to convert ${\tilde{z}}^{\left(i\right)}∼N\left(\mu^{\left(i\right)},{\left(\sigma +\xi \right)}^{2(i)}\right)$ to ${\tilde{z}}^{\left(i\right)}=\mu^{\left(i\right)}+{\left(\sigma +\xi \right)}^{\left(i\right)}\epsilon ,\;\epsilon ∼N\left(0,I\right)$ to make ξ differentiable. In summary, the formal loss function $L_{Indicator}$ is expressed as follows:

(8) $$\begin{array}{cc} L_{indicator}\left(\beta \right)= & E_{p(x)}\left[E_{q\left(z|x\right),\;\tilde{q}\left(\tilde{z}|x\right)}\left[L2\left(TM\left(Dec\left(z\right)\right),TM\left(Dec\left(\tilde{z}\right)\right)\right)\right]\right]-\lambda \sum_{i=1}^{B}f\left(\beta_{i}\right)+{∥\beta ∥}_{2},\\ q(z|x)=N(\mu , & \sigma^{2}),\;\tilde{q}\left(\tilde{z}|x\right)=N\left(\mu ,{\left(\sigma +f\left(\beta \right)\right)}^{2}\right) \end{array}$$

Finally, the representation dimensions corresponding to the parameters satisfying $|\beta_{i}|<\psi$ are considered more relevant by the task model and their indices are recorded, where ψ is the threshold. The entire training process of the proposed Indicator is shown in Algorithm 1.
**Algorithm 1** Indicator Training1:**Input:** Encoder Enc(), Conversion function f(), Decoder Dec(), task model Tm(), disentangled representation size *B*, threshold ψ.2:**Output:** Dimension index to be retained Index.3:β← random initialize Indicator parameters4:**for** epochs **do**5:   Random mini-batch $X=\{x^{\left(1\right)},x^{\left(2\right)},\cdots ,x^{\left(n\right)}\}$,6:   $\mu ,\sigma =Enc\left(X\right),\mu ,\sigma \in R^{n\times B}$7:   z=μ+σ×ϵ,ϵ∼N(0,I)8:   $\tilde{z}=\mu +\left(\sigma +f\left(\beta \right)\right)\times \epsilon ,\;\epsilon ∼N\left(0,I\right)$9:   $l=Tm\left(Dec\left(z\right)\right),\;\tilde{l}=Tm\left(Dec\left(\tilde{z}\right)\right)$10:   $\beta \overset{update}{⟵}min\left(L2\left(l,\tilde{l}\right)-\sum_{i=1}^{B}f\left(\beta_{i}\right)\right)+{∥\beta ∥}_{2}$11:**end for**12:Index={}13:**for** $\beta_{i}\in \beta$ **do**14:   **if** $|\beta_{i}|<\psi$ **then**15:     Index.append(i)16:   **end if**17:**end for**

### 3.4. Data Reconstruction

The disentangled representation encoded by the encoder allows obfuscation of the task-independent features without changing the task-related features. It is necessary to preserve the *m* dimensions marked by Indicator because the task model pays more attention to them. At the same time, the remaining B−m dimensions, which contribute less to task inference but contain excessive task-independent information, should be discarded. Theoretically, it is possible to replace the original values of the B−m dimensions with arbitrary values. In practice, however, completely random values will make it easier for the transformed representation to decode ambiguous data, resulting in task-relevant information not being correctly expressed. Even though the B−m dimensions have nothing to do with the task model, it is still necessary to be careful when choosing their replacement values. As shown in the test phase of Figure 4, our method uses an arbitrary sample as a carrier. It concatenates the B−m dimensions in the carrier representation with the *m* dimensions in the original data representation. By reconstructing the data from such a representation, only the factors that the task model focuses on are credible, while others are confusing.

## 4. Experimental Study

In this section, we first qualitatively evaluate the proposed Indicator and report the experimental results. Then, we quantify the privacy–utility trade-offs of our framework and present a comparison with other popular methods. The following experiments involve three datasets: dSprites [47], MNIST [48], and CelebA [49]. dSprites contains 737,280 2D synthesis samples with 6 attributes. We randomly divide 589,824 samples for training VAE family models and Indicators and 147,456 samples for testing. MNIST contains grayscale images of 10 classes of handwritten digits, including 60,000 training samples and 10,000 testing samples. CelebA includes 202,599 face images labeled with 40 binary attributes, of which 162,770 images are divided for training and 39,829 images for testing. The experiments are conducted on Nvidia GTX 3080Ti GPU in Pytorch.

### 4.1. Indicator Evaluation

To qualitatively demonstrate the effectiveness of the proposed Indicator, we conduct experiments on dSprites and MNIST from three perspectives. (a) Versatility: whether the proposed Indicator can be effectively combined with the various VAEs models. (b) Reliability: whether the task model considers the dimensions marked by Indicator. (c) Stability: whether the Indicator can make the same decision under different initial conditions and training subsets.

#### 4.1.1. Versatility

To illustrate the versatility, the following experiments are performed on β-VAE, Factor-VAE, and β-TCVAE, respectively.

#### 4.1.2. Reliability

The verification of the reliability is studied by two experiments. For dSprites and MNIST, the dimension *B* of the disentangled representation is set to 10, and the threshold δ is set to 0.5. For dSprites, a classifier focusing on the X-position is used as the target task model. For β-VAE, Factor-VAE, and β-TCVAE, Indicator finds 3, 4, and 2 dimensions on which the target task model focuses. For MNIST, a classifier that distinguishes digits is the target task model. In the above three VAE models, Indicator finds 3, 4, and 3 task-related dimensions in the representation.

**Figure 6:**
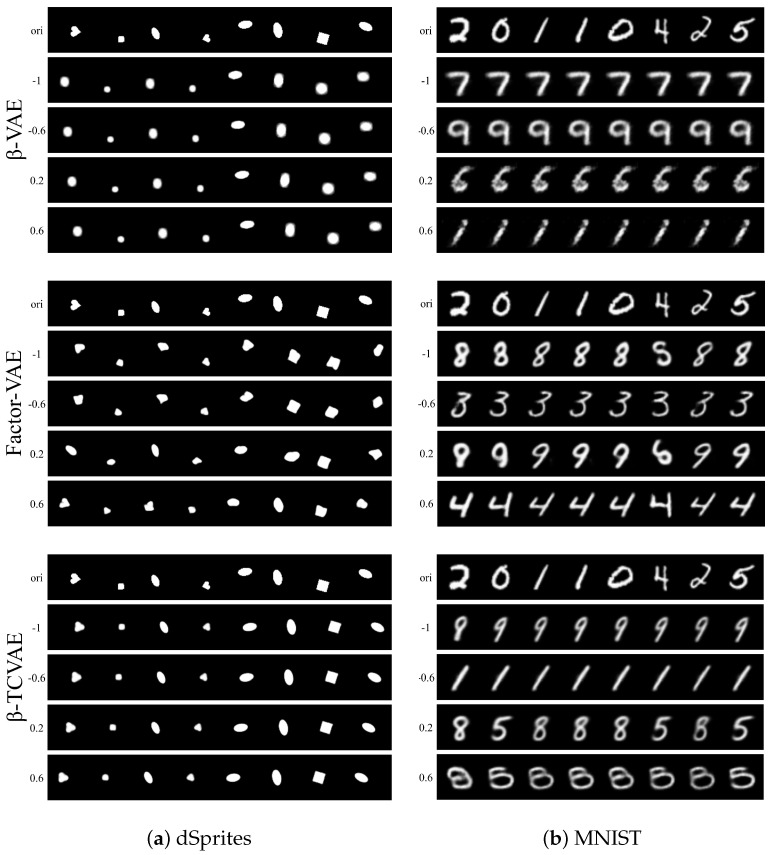
Reconstructed image visualization of traversing the representation dimensions marked by the indicator.

**Figure 7:**
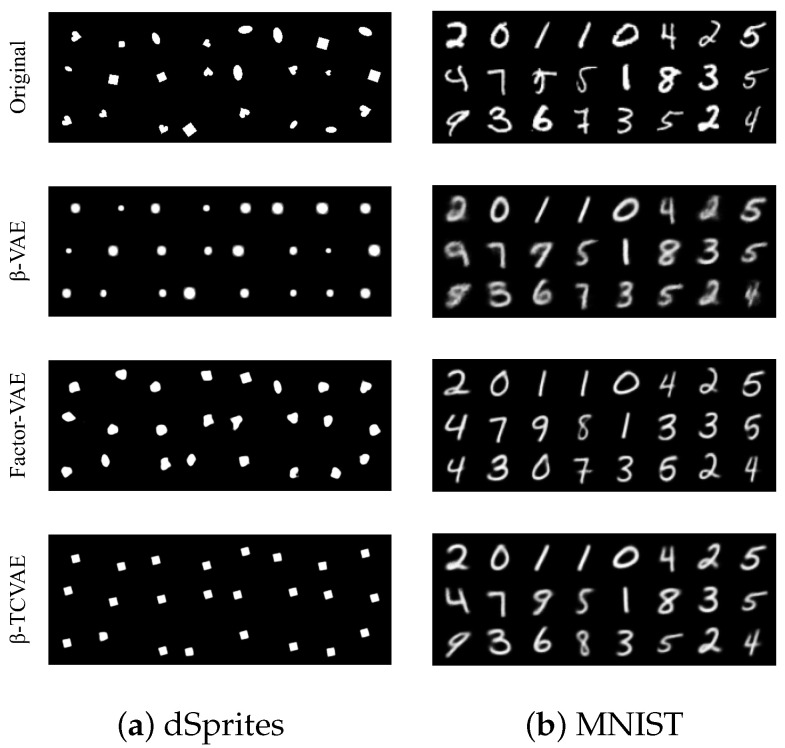
The representation dimensions marked by Indicator are fixed, while the values of the remaining dimensions are replaced with 0. The above illustrations are reconstructed images based on these processed representations.

The first experiment is to interpolate the dimensions marked by Indicator while freezing the remaining dimensions. Figure 6 visualizes the reconstructed image traversing the marked dimensions. The first line is the original data, and the second to fifth lines are the reconstruction of the interpolated representation. On the one hand, the “X-position” that the task model focuses on changes with the change in the marked representation dimension. On the other hand, the experimental results show the difference in disentangling performance of different VAE schemes. In the second experiment, we fix the dimensions marked by Indicator and replace the values of the remaining dimensions with 0. Figure 7 shows the reconstruction of the processed representation. The attribute focused by the target task model is preserved, while the others become irrelevant to the original data. The above two experiments show that the task-related dimensions determined by the proposed Indicator are consistent with the human view, which confirms the reliability of Indicator to a certain extent. The quantitative measure of reliability can be decomposed into target task accuracy and the availability of task-independent attributes. Target task accuracy reflects whether the task-related dimensions are fully selected. The availability of task-independent attributes is directly proportional to the redundancy of the selected dimensions. Therefore, reliability is equivalent to the privacy–utility trade-offs of our framework, which will be discussed on the CelebA dataset in Section 4.2.

#### 4.1.3. Stability

**Figure 8:**
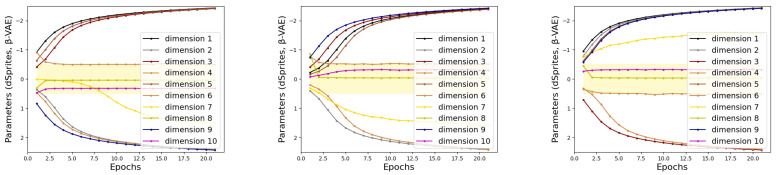

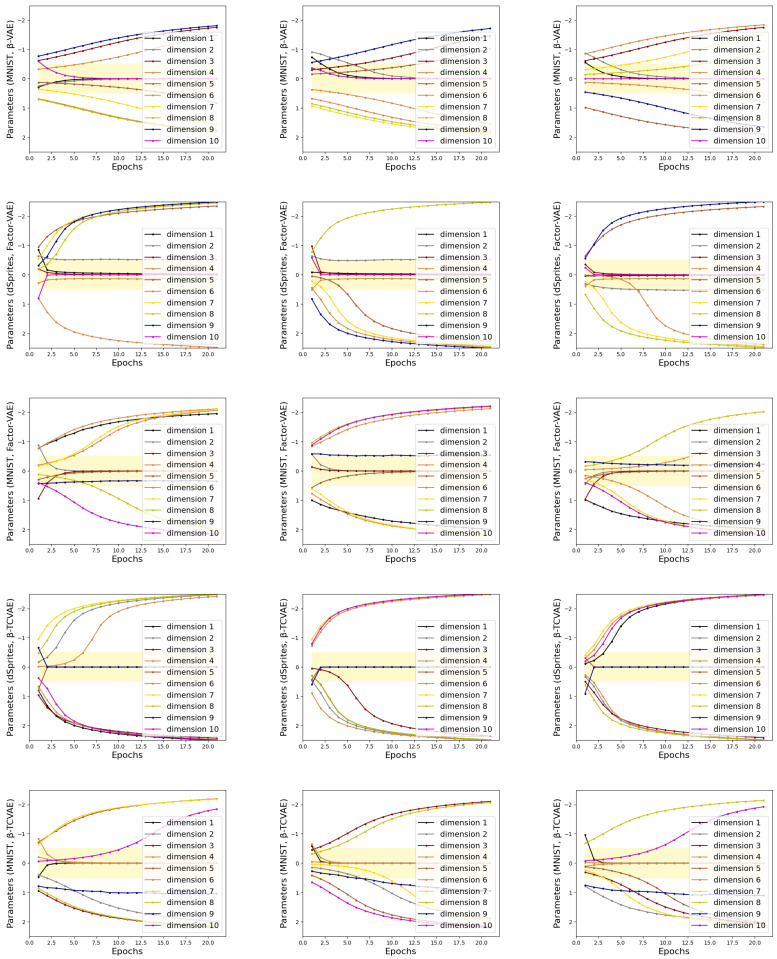
The training sets of dSprites and MNIST are divided into 3 subsets, respectively. The above illustration is the parameter curve obtained by Indicator training on these subsets. In the illustration, the dimensions that fall into the yellow area are considered more concerned by the task model. In rows 1 and 2, the indicators mark the disentangled representations generated from β-VAE. Indicators in lines 3 and 4 mark the disentangled representations generated using Factor-VAE. Lines 5 and 6 are Indicator marking the disentangled representations generated using β-TCVAE.

To illustrate the stability of the proposed Indicator, we perform experiments on dSprites and MNIST with the same settings as in Section 4.1.2. The train sets of dSprites and MNIST are divided into 3 subsets, and then Indicator searches for task-related dimensions on each subset with random initial parameters. Figure 8 provides a visualization of Indicator parameters changing with epochs. In Figure 8, the dimensions that fall into the yellow area are considered more concerned by the task model. In rows 1 and 2, the indicators mark the disentangled representations generated from β-VAE. The Indicators in lines 3 and 4 mark the disentangled representations generated using Factor-VAE. Lines 5 and 6 are Indicator marking the disentangled representations generated using β-TCVAE. Taking the three subfigures in the first row as an example, the parameters corresponding to dimensions 4, 8, and 10 in the Indicator eventually converge to the yellow region, while the rest diverge. This represents that Indicator considers dimensions 4, 8 and 10 as being attended to by the target task. Under different dataset slices and random initial parameters, the tendency of Indicators to represent dimensions shows the same trend. This demonstrates the stability of the Indicator, where the marking process is not affected by the initial parameters and the division of the dataset. Moreover, the experimental results also support the conclusion that the same dimensions of the latent code of different samples correspond to the same information.

### 4.2. Privacy–Utility Trade-Offs Evaluation and Comparison

#### 4.2.1. Setup

We design experiments to verify the effectiveness of our framework’s utility–privacy trade-offs on the real-world dataset CelebA. The images are normalized and resized to 3×64×64 for preprocessing. Due to the better disentanglement of β-TCVAE, the β-TCVAE optimized by GAN is chosen to construct our partial privacy-preserving framework. The encoder and decoder are optimized using RmSprop, with alpha and eps set to 0.9 and $1\times 10^{-8}$, respectively. The discriminator is trained using an SGD optimizer, with momentum and weight_decay set to 0.9 and $1\times 10^{-4}$, respectively. We train these three components for 40 epochs with a fixed learning rate of $3\times 10^{-4}$, and the batch_size is set to 128. The dimension size of the disentangled representation is set to 128, which represents the output of the encoder, including 128 means and 128 variances. Indicator is trained using the SGD optimizer with 0.9momentum and $1\times 10^{-4}$weight_decay for 20 epochs with batch_size 256. The learning rate is set to $1\times 10^{-4}$. Empirically, we set the hyperparameter λ to 2 and δ to 1. The classifier trained on the original data with the standard ResNet18 architecture is considered the task model.

In our experiments, the accuracy of the task model is used to quantify utility. Several attack models designed to infer privacy attributes are introduced, and we propose two new metrics as privacy measures. The data processed by the task model and the attack models come from the reconstruction of the decoder. Despite the introduction of GAN, the reconstructed data still inevitably loses details. In order to avoid exaggerating the protective effect of our privacy attribute framework due to the fuzziness of the reconstruction, we set the easily recognizable attributes as the platform for the privacy utility measurement. Specifically, we set “Eyeglasses” and “Gender” as the target attributes of the task model, while enumerating “Wearing_Hat” and “Bald” as the target for the attack models.

#### 4.2.2. Baselines

We choose three classical privacy-preserving schemes that are widely used in the literature as a baseline against which to compare our framework. A brief description of these schemes is given below. Gaussian noise obfuscates the raw data by adding Gaussian noise $N(0,\sigma^{2})$, where σ is set to 0.5 and 1, respectively. Because Gaussian noise can provide rigorous differential privacy guarantees with less local noise, it is widely used in federated learning scenarios [30,50]. Laplacian noise is also a classic differential privacy method that injects Laplacian noise into the raw data according to the privacy budget {0.3,0.9}. PAN is a representative framework for adversarial training methods [13]. In the training phase, PAN simulates adversaries interested in private information to obtain an encoder that can extract the representation with good utility–privacy trade-offs. In the comparison phase, the objective function adopts two sets of coefficients {0.1,0.7,0.2}, {0.5,0.3,0.2} to show its performance under different privacy budgets.

#### 4.2.3. Evaluation and Comparison

To quantify the data utility maintained by different schemes, the classification accuracy of the target attribute is measured. Specifically, the two noise injection methods and our framework use classifiers trained on the raw dataset, while PAN uses the utility discriminator generated in the adversarial training. In terms of privacy measurement, the accuracy of the adversarial model’s inference of privacy attributes is not convincing. This is because the model’s decision is biased and the test set samples may be uneven, which means that lower accuracy does not necessarily mean better privacy protection. Taking the “bald” attribute as an example, the uniform random noise with a value in the range [0,1] will be 100% judged as not bald by the adversary model with an average confidence of 0.99. Although these noises are unrelated to the raw data, they will still achieve 97.88% inference accuracy when considered as a processed private image. In addition, the confidence difference in the attack models in inferring private images will reveal additional information compared to inferring random noise. Therefore, we propose the average confidence difference *Con-Diff* and the distribution shift *Dis-Shift* as the privacy quantification. The two formulas are defined as follows:

(9) $$\begin{array}{c} Con\_Diff=\frac{1}{N}\sum_{i=1}^{N}\left(AM^{l}{\left({x^{\prime }}^{\left(i\right)}\right)}^{\left(0\right)}-AM^{l}{\left(noise^{\left(i\right)}\right)}^{\left(0\right)}\right) \end{array}$$

(10) $$\begin{array}{c} Dis\_Shift=\frac{1}{N}\sum_{i=1}^{N}\left|AM\left({x^{\prime }}^{\left(i\right)}\right)-AM\left(noise^{\left(i\right)}\right)\right| \end{array}$$

Among them, AM is the attack model with *l* layers, $x^{\prime }$ represents private images generated by different methods, and *N* is the total number of samples in the test set. $AM^{l}{\left(\cdot \right)}^{\left(0\right)}$ indicates the first element output by the AM, and AM(·) represents class 0 or 1. The lower *Con-Diff* and *Dis-Shift* represent that the attack model’s inference on the processed data privacy attributes is closer to a non-priority guess, which demonstrates better privacy considerations.

**Figure 9:**
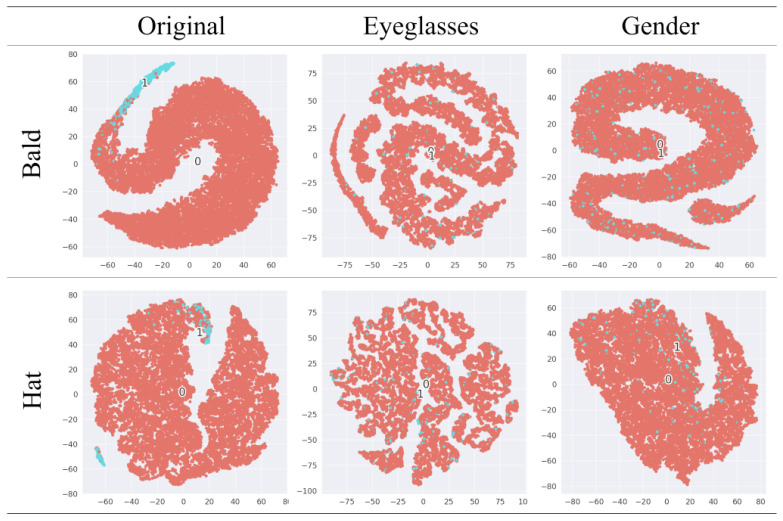
The t-SNE visualization of the $AM^{l-1}$ output. The first column represents the performance of the original data in the face of the attack model. The second and third columns are the anonymously transformed reconstruction performance facing the attack model, with “Eyeglasses” and “Gender” as the task-related attributes, respectively.

In Figure 9, we use t-SNE [51] to visualize the features learned by the attack model at layer l-1 to analyze the effectiveness of our framework. The first column is the t-SNE plot of the original image facing the attack model. The second and third columns show the t-SNE plot for anonymously transformed reconstruction with “Eyeglasses” and “Gender” as the task-related attributes. The original data features show significant clustering for the two task-independent attributes, while the features of anonymously transformed reconstruction are indistinguishable.

**Figure 10:**
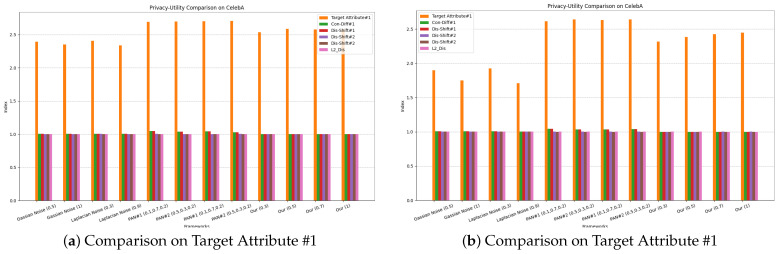
Privacy–utility comparison on CelebA. Among them, the y-axis takes the exp(·) of the evaluation result.

**Figure 11:**
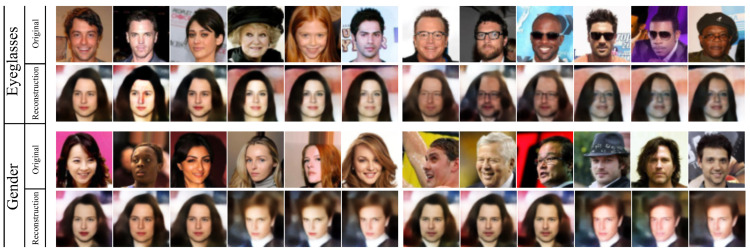
The above illustrations are facial images whose task-independent attributes are confused. The upper part takes “Eyeglasses” as the task-related attribute, and the bottom part, “Gender” is regarded as the task-related attribute.

Table 1 shows the evaluation and comparison of different methods on the utility–privacy trade-offs. It also includes L2 distance to measure the similarity between the processed image and the original data. “Target Attribute #1” and “Target Attribute #2” represent “Eyeglasses” and “Gender”, while “Privacy Attribute #1” and “Privacy Attribute #2” represent “Wearing_Hat” and “Bald”. Injecting Gaussian noise and Laplacian noise are general methods that will affect all attributes indiscriminately. Therefore, the privacy protection of these methods will significantly sacrifice its utility. In addition, L2-DIS indicate that the processed image still has a high similarity to the original data, which will also lead to the risk of privacy leakage. For the evaluation of PAN, we follow its recommendation on the encoder structure and design 4 convolutional layers, 4 normalization layers, 2 Maxpooling, and 2 upsampling layers. After 15 epochs of training, PAN achieves the ideal utility, but more discussion about privacy is necessary. In our experiment, the attack model for privacy attribute #1 will classify uniform random noise as class “1” with 100% probability, and 3.3% of the samples in the test set are class “1”. At the same time, the attack model for privacy attribute #2 will infer uniform random noise as class “0” with 100% probability, and 97.9% of the test set are “0” samples. Under different experimental settings, some samples fool the attack model’s judgment on privacy attribute #1, but have little effect on privacy attribute #2. Similarly, for these two privacy attributes, the attack model’s judgments on the encrypted data are closer to its judgments on random noise. The mechanism of classifiers based on neural networks can be simply described as being oriented to data distribution. The nonlinear transformation of the original data by the encoder in PAN essentially causes a distribution shift. From our point of view, this is the privacy guarantee of PAN. However, there are still a large number of samples that reveal the privacy attributes. The evaluation of our framework uses the inference results of the attack model on the carrier as a benchmark. While the reconstructed image retains utility, there is almost no difference in confidence and distribution shift compared to the carrier. This shows that the reconstructed image produces a low-level information gain for the attack model, demonstrating the privacy of our framework. In order to compare different frameworks more intuitively, we further describe the evaluation results in Figure 10. The visualization of the reconstructed images shown in Figure 11 supports the evaluation results. The upper part of each sub-region in Figure 9 are the original images, and the lower part are the reconstructed images, which retain the target attribute while others still belong to the carrier. Since the reconstructed images are also facial images, the structure is similar to the original image. Another advantage of our framework is its flexibility. When the target or privacy attributes change, our framework needs to retrain 128 parameters, but PAN needs to retrain 22.44 M.

Using the privacy attribute #1 as a platform, we further explore potential attackers against the baseline methods and our framework. More powerful attack models are trained using the privacy-edited data as input and combining the ground truth. It should be noted that these attacks may not be feasible in real-world scenarios, and we aim to explore whether the above methods can effectively confuse the original data. The experimental results are reported in Table 2. The new attack models do not perform more effective attacks against the two noise injection methods and our framework, which shows that the topological space of the original data is broken. On the contrary, PAN is vulnerable to new attacks, supporting the suspicion mentioned in Section 1.

To further show the effectiveness of our framework, we also use SVM for experiments. We choose the RBF kernel function, use libsvm to set the hyperparameters and let the latent code be the input. Evaluation results are shown in Table 3.

Based on the above experimental results, on the one hand, it can be observed that our framework can be more effective against potential attackers compared to adversarial training based PAN. On the other hand, our framework maintains better data availability as well as privacy of task-independent attributes compared to the noise adding approach.

## 5. Discussion and Conclusions

In this work, we design an Indicator to indicate the region of interest of the target task model on the disentangled representation. By retaining the information necessary for the target task through Indicator, we further construct a privacy-preserving prediction framework that respects the task-independent attributes. Evaluations on multiple standard datasets show that our framework achieves competitive utility–privacy trade-offs.

However, our framework has not yet reached the ideal situation of preserving all utility and protecting all privacy. On the one hand, our framework partially loses accuracy in the target task. On the other hand, the attacker’s accuracy in inferring privacy attributes is higher than the guess without prior knowledge. We speculate that there are two reasons: (a) the quality of the reconstructed image limits the utility; (b) there is information overlap between the different representation dimensions, leading to sensitive information leakage. These are also problems that we hope to solve in the future.

## Figures and Tables

**Table 1:** Privacy–utility comparison on CelebA.

Methods	Target Attribute #1	Privacy Attribute #1		Privacy Attribute #2		L2-DIS
		**Dis-Shift**	**Con-Diff**	**Dis-Shift**	**Con-Diff**	
Gaussian noise $\left(\sigma^{2}=0.5\right)$	87.3%	2.45%	$6.11\times 10^{-2}$	0.01%	$1.90\times 10^{-2}$	$6.19\times 10^{-4}$
Gaussian noise $\left(\sigma^{2}=1\right)$	85.5%	0.31%	$5.05\times 10^{-2}$	0.02%	$1.85\times 10^{-2}$	$8.04\times 10^{-4}$
Laplacian noise (λ=0.3)	87.8%	12.8%	$5.51\times 10^{-2}$	0.03%	$1.91\times 10^{-2}$	$5.82\times 10^{-4}$
Laplacian noise (λ=0.9)	85.0%	3.53%	$3.49\times 10^{-2}$	0.01%	$1.80\times 10^{-2}$	$8.06\times 10^{-4}$
PAN (#1, (0.1,0.7,0.2))	99.1%	50.7%	$4.72\times 10^{-1}$	0.05%	$1.33\times 10^{-4}$	$1.52\times 10^{-3}$
PAN (#1, (0.5,0.3,0.2))	99.3%	37.3%	$3.53\times 10^{-1}$	0.02%	$1.89\times 10^{-4}$	$1.95\times 10^{-3}$
PAN (#2, (0.1,0.7,0.2))	99.4%	41.4%	$4.12\times 10^{-1}$	0.02%	$3.49\times 10^{-4}$	$1.26\times 10^{-3}$
PAN (#2, (0.5,0.3,0.2))	99.5%	16.1%	$2.73\times 10^{-1}$	0.05%	$3.18\times 10^{-4}$	$1.69\times 10^{-3}$
**Our framework** (ψ=0.3)	93.1%	0%	$1.32\times 10^{-6}$	0%	0	$9.86\times 10^{-4}$
**Our framework** (ψ=0.5)	95.0%	0%	$1.81\times 10^{-6}$	0%	0	$8.96\times 10^{-4}$
**Our framework** (ψ=0.7)	94.6%	0%	$1.44\times 10^{-6}$	0%	0	$8.44\times 10^{-4}$
**Our framework** (ψ=1)	94.6%	0%	$3.15\times 10^{-6}$	0%	$2.60\times 10^{-10}$	$7.52\times 10^{-4}$
**Methods**	**Target Attribute #2**	**Privacy Attribute #1**		**Privacy Attribute #2**		**L2-DIS**
		**Dis-Shift**	**Con-Diff**	**Dis-Shift**	**Con-Diff**	
Gaussian noise $\left(\sigma^{2}=0.5\right)$	64.0%	2.45%	$6.11\times 10^{-2}$	0.01%	$1.90\times 10^{-2}$	$6.19\times 10^{-2}$
Gaussian noise $\left(\sigma^{2}=1\right)$	55.8%	0.31%	$5.05\times 10^{-2}$	0.02%	$1.85\times 10^{-2}$	$8.04\times 10^{-4}$
Laplacian noise (λ=0.3)	65.4%	12.8%	$5.51\times 10^{-2}$	0.03%	$1.91\times 10^{-2}$	$5.82\times 10^{-4}$
Laplacian noise (λ=0.9)	53.7%	3.53%	$3.49\times 10^{-2}$	0.01%	$1.80\times 10^{-2}$	$8.06\times 10^{-4}$
PAN (#1, (0.1,0.7,0.2))	96.1%	46.9%	$4.47\times 10^{-1}$	0.15%	$2.20\times 10^{-4}$	$1.05\times 10^{-3}$
PAN (#1, (0.5,0.3,0.2))	97.0%	43.3%	$3.34\times 10^{-1}$	0.22%	$3.69\times 10^{-4}$	$1.05\times 10^{-3}$
PAN (#2, (0.1,0.7,0.2))	96.8%	28.5%	$3.45\times 10^{-1}$	0.27%	$2.28\times 10^{-4}$	$1.06\times 10^{-3}$
PAN (#2, (0.5,0.3,0.2))	97.0%	35.6%	$3.93\times 10^{-1}$	0.15%	$2.45\times 10^{-4}$	$1.05\times 10^{-3}$
**Our framework** (ψ=0.3)	84.0%	0.02%	$1.65\times 10^{-6}$	0%	0	$7.41\times 10^{-4}$
**Our framework** (ψ=0.5)	86.9%	0.03%	$1.60\times 10^{-6}$	0%	0	$5.56\times 10^{-4}$
**Our framework** (ψ=0.7)	88.6%	0.05%	$4.09\times 10^{-6}$	0.12%	$1.03\times 10^{-7}$	$3.29\times 10^{-4}$
**Our framework** (ψ=1)	89.5%	0.78%	$1.61\times 10^{-4}$	0.40%	$2.92\times 10^{-5}$	$2.21\times 10^{-4}$

**Table 2:** Search for potential attacker.

Methods	Dis-Shift (Original Attacker)	Dis-Shift (Potential Attacker)
Gaussian noise $\left(\sigma^{2}=0.5\right)$	2.45%	3.76%
Gaussian noise $\left(\sigma^{2}=1\right)$	0.31%	3.51%
Laplacian noise (λ=0.3)	12.8%	4.44%
Laplacian noise (λ=0.9)	3.53%	3.14%
PAN (0.5,0.3,0.2)	43.3%	95.52%
PAN (0.1,0.7,0.2)	46.9%	96.74%
**Our framework** (ψ=0.5)	0.03%	0.05%
**Our framework** (ψ=1)	0.78%	0.91%

**Table 3:** Evaluating privacy–utility on CelebA via SVM.

Method	Target Attribute #1	Privacy Attribute #1	Privacy Attribute #2
Our framework (ψ=0.3)	84.3%	43.1%	54.2%
Our framework (ψ=0.5)	85.9%	46.3%	55.6%
Our framework (ψ=0.7)	87.2%	48.4%	56.9%
Our framework (ψ=1)	87.5%	50.3%	58.4%
**Method**	**Target Attribute #1**	**Privacy Attribute #1**	**Privacy Attribute #2**
Our framework (ψ=0.3)	72.5%	44.5%	54.6%
Our framework (ψ=0.5)	73.2%	47.8%	55.8%
Our framework (ψ=0.7)	73.7%	47.7%	57.2%
Our framework (ψ=1)	74.1%	48.4%	59.1%

## Data Availability

Our experiments use the public datasets MNIST, dSprites and CelebA.

## References

[1] Devlin J., Chang M., Lee K., Toutanova K., Burstein J., Doran C., Solorio T. (2019). BERT: Pre-training of Deep Bidirectional Transformers for Language Understanding. Proceedings of the 2019 Conference of the North American Chapter of the Association for Computational Linguistics: Human Language Technologies, NAACL-HLT 2019.

[2] Dosovitskiy A., Beyer L., Kolesnikov A., Weissenborn D., Zhai X., Unterthiner T., Dehghani M., Minderer M., Heigold G., Gelly S. (2020). An Image is Worth 16x16 Words: Transformers for Image Recognition at Scale. arXiv.

[3] Li A., Guo J., Yang H., Salim F.D., Chen Y. (2021). DeepObfuscator: Obfuscating Intermediate Representations with Privacy-Preserving Adversarial Learning on Smartphones. Proceedings of the IoTDI’21: International Conference on Internet-of-Things Design and Implementation.

[4] Ribeiro M., Grolinger K., Capretz M.A. MLaaS: Machine Learning as a Service. Proceedings of the 2015 IEEE 14th International Conference on Machine Learning and Applications (ICMLA).

[5] Achille A., Soatto S. (2018). Emergence of Invariance and Disentanglement in Deep Representations. J. Mach. Learn. Res..

[6] Google (2018). Google Now Launcher. https://en.wikipedia.org/wiki/Google_Now.

[7] Google (2018). Data Preparation. https://cloud.google.com/ml-engine/docs/tensorflow/data-prep.

[8] Fredrikson M., Jha S., Ristenpart T. Model Inversion Attacks That Exploit Confidence Information and Basic Countermeasures. Proceedings of the CCS’15, 22nd ACM SIGSAC Conference on Computer and Communications Security.

[9] Mahendran A., Vedaldi A. Understanding deep image representations by inverting them. Proceedings of the 2015 IEEE Conference on Computer Vision and Pattern Recognition (CVPR).

[10] Hidano S., Murakami T., Katsumata S., Kiyomoto S., Hanaoka G. Model Inversion Attacks for Prediction Systems: Without Knowledge of Non-Sensitive Attributes. Proceedings of the 2017 15th Annual Conference on Privacy, Security and Trust (PST).

[11] Osia S.A., Shahin Shamsabadi A., Sajadmanesh S., Taheri A., Katevas K., Rabiee H.R., Lane N.D., Haddadi H. (2020). A Hybrid Deep Learning Architecture for Privacy-Preserving Mobile Analytics. IEEE Internet Things J..

[12] Goodfellow I., Pouget-Abadie J., Mirza M., Xu B., Warde-Farley D., Ozair S., Courville A., Bengio Y., Ghahramani Z., Welling M., Cortes C., Lawrence N., Weinberger K.Q. (2014). Generative Adversarial Nets. Advances in Neural Information Processing Systems.

[13] Liu S., Du J., Shrivastava A., Zhong L. (2019). Privacy Adversarial Network: Representation Learning for Mobile Data Privacy. Proc. Acm Interact. Mobile, Wearable Ubiquitous Technol..

[14] Li A., Duan Y., Yang H., Chen Y., Yang J. TIPRDC: Task-Independent Privacy-Respecting Data Crowdsourcing Framework for Deep Learning with Anonymized Intermediate Representations. Proceedings of the KDD’20, 26th ACM SIGKDD International Conference on Knowledge Discovery & Data Mining.

[15] Zhou B., Khosla A., Lapedriza A., Oliva A., Torralba A. Learning Deep Features for Discriminative Localization. Proceedings of the 2016 IEEE Conference on Computer Vision and Pattern Recognition (CVPR).

[16] Chattopadhay A., Sarkar A., Howlader P., Balasubramanian V.N. Grad-CAM++: Generalized Gradient-Based Visual Explanations for Deep Convolutional Networks. Proceedings of the 2018 IEEE Winter Conference on Applications of Computer Vision (WACV).

[17] Zhang Q., Rao L., Yang Y. (2021). Group-CAM: Group Score-Weighted Visual Explanations for Deep Convolutional Networks. arXiv.

[18] Zhang Q., Wang X., Wu Y.N., Zhou H., Zhu S.C. (2021). Interpretable CNNs for Object Classification. IEEE Trans. Pattern Anal. Mach. Intell..

[19] Higgins I., Matthey L., Pal A., Burgess C., Glorot X., Botvinick M., Mohamed S., Lerchner A. beta-VAE: Learning Basic Visual Concepts with a Constrained Variational Framework. Proceedings of the 5th International Conference on Learning Representations, ICLR 2017.

[20] Kim H., Mnih A., Dy J.G., Krause A. (2018). Disentangling by Factorising. Proceedings of the 35th International Conference on Machine Learning, ICML 2018, Stockholmsmässan.

[21] Chen T.Q., Li X., Grosse R.B., Duvenaud D., Bengio S., Wallach H.M., Larochelle H., Grauman K., Cesa-Bianchi N., Garnett R. (2018). Isolating Sources of Disentanglement in Variational Autoencoders. Advances in Neural Information Processing Systems 31, Proceedings of theAnnual Conference on Neural Information Processing Systems 2018, NeurIPS 2018, Montréal, QC, Canada, 3–8 December 2018.

[22] Chen X., Duan Y., Houthooft R., Schulman J., Sutskever I., Abbeel P., Lee D.D., Sugiyama M., von Luxburg U., Guyon I., Garnett R. (2016). InfoGAN: Interpretable Representation Learning by Information Maximizing Generative Adversarial Nets. Advances in Neural Information Processing Systems 29, Proceedings of theAnnual Conference on Neural Information Processing Systems 2016, Barcelona, Spain, 5–10 December 2016.

[23] Kingma D.P., Dhariwal P., Bengio S., Wallach H.M., Larochelle H., Grauman K., Cesa-Bianchi N., Garnett R. (2018). Glow: Generative Flow with Invertible 1x1 Convolutions. Advances in Neural Information Processing Systems 31, Proceedings of the Annual Conference on Neural Information Processing Systems 2018, NeurIPS 2018, Montréal, QC, Canada 3–8 December 2018.

[24] Sweeney L. (2002). k-Anonymity: A Model for Protecting Privacy. Int. J. Uncertain. Fuzziness Knowl. Based Syst..

[25] Machanavajjhala A., Gehrke J., Kifer D., Venkitasubramaniam M. L-diversity: Privacy beyond k-anonymity. Proceedings of the 22nd International Conference on Data Engineering (ICDE’06).

[26] Li N., Li T., Venkatasubramanian S. t-Closeness: Privacy Beyond k-Anonymity and l-Diversity. Proceedings of the 2007 IEEE 23rd International Conference on Data Engineering.

[27] Dwork C., Roth A. (2014). The Algorithmic Foundations of Differential Privacy. Found. Trends Theor. Comput. Sci..

[28] Mironov I. Rényi Differential Privacy. Proceedings of the 2017 IEEE 30th Computer Security Foundations Symposium (CSF).

[29] Abadi M., Chu A., Goodfellow I., McMahan H.B., Mironov I., Talwar K., Zhang L. Deep Learning with Differential Privacy. Proceedings of the CCS’16, 2016 ACM SIGSAC Conference on Computer and Communications Security.

[30] Papernot N., Song S., Mironov I., Raghunathan A., Talwar K., Erlingsson Ú. Scalable Private Learning with PATE. Proceedings of the 6th International Conference on Learning Representations, ICLR 2018.

[31] Oh S.J., Benenson R., Fritz M., Schiele B., Leibe B., Matas J., Sebe N., Welling M. (2016). Faceless Person Recognition: Privacy Implications in Social Media. Computer Vision—ECCV 2016.

[32] Dowlin N., Gilad-Bachrach R., Laine K., Lauter K., Naehrig M., Wernsing J. CryptoNets: Applying Neural Networks to Encrypted Data with High Throughput and Accuracy. Proceedings of the ICML’16 33rd International Conference on International Conference on Machine Learning.

[33] Li J., Kuang X., Lin S., Ma X., Tang Y. (2020). Privacy preservation for machine learning training and classification based on homomorphic encryption schemes. Inf. Sci..

[34] Riazi M.S., Weinert C., Tkachenko O., Songhori E.M., Schneider T., Koushanfar F. Chameleon: A Hybrid Secure Computation Framework for Machine Learning Applications. Proceedings of the ASIACCS’18, 2018 on Asia Conference on Computer and Communications Security.

[35] Liu J., Juuti M., Lu Y., Asokan N. Oblivious Neural Network Predictions via MiniONN Transformations. Proceedings of the CCS’17, Proceedings of the 2017 ACM SIGSAC Conference on Computer and Communications Security.

[36] Mohassel P., Zhang Y. SecureML: A System for Scalable Privacy-Preserving Machine Learning. Proceedings of the 2017 IEEE Symposium on Security and Privacy (SP).

[37] Yu J., Zhang B., Kuang Z., Lin D., Fan J. (2017). iPrivacy: Image Privacy Protection by Identifying Sensitive Objects via Deep Multi-Task Learning. IEEE Trans. Inf. Forensics Secur..

[38] Malekzadeh M., Clegg R.G., Haddadi H. Replacement AutoEncoder: A Privacy-Preserving Algorithm for Sensory Data Analysis. Proceedings of the 2018 IEEE/ACM Third International Conference on Internet-of-Things Design and Implementation (IoTDI).

[39] Aloufi R., Haddadi H., Boyle D. Privacy-Preserving Voice Analysis via Disentangled Representations. Proceedings of the CCSW’20, 2020 ACM SIGSAC Conference on Cloud Computing Security Workshop.

[40] van den Oord A., Vinyals O., kavukcuoglu K., Guyon I., Luxburg U.V., Bengio S., Wallach H., Fergus R., Vishwanathan S., Garnett R. (2017). Neural Discrete Representation Learning. Advances in Neural Information Processing Systems.

[41] Kalchbrenner N., Elsen E., Simonyan K., Noury S., Casagrande N., Lockhart E., Stimberg F., van den Oord A., Dieleman S., Kavukcuoglu K., Dy J.G., Krause A. (2018). Efficient Neural Audio Synthesis. Proceedings of the 35th International Conference on Machine Learning, ICML 2018, Stockholmsmässan.

[42] Gong M., Liu J., Li H., Xie Y., Tang Z. (2020). Disentangled Representation Learning for Multiple Attributes Preserving Face Deidentification. IEEE Trans. Neural Netw. Learn. Syst..

[43] Wu H., Tian X., Li M., Liu Y., Ananthanarayanan G., Xu F., Zhong S. PECAM: Privacy-Enhanced Video Streaming and Analytics via Securely Reversible Transformation. Proceedings of the MobiCom’21, 27th Annual International Conference on Mobile Computing and Networking.

[44] Jia J., Gong N.Z. Attriguard: A Practical Defense against Attribute Inference Attacks via Adversarial Machine Learning. Proceedings of the SEC’18, 27th USENIX Conference on Security Symposium.

[45] Wu Z., Wang Z., Wang Z., Jin H., Ferrari V., Hebert M., Sminchisescu C., Weiss Y. (2018). Towards Privacy-Preserving Visual Recognition via Adversarial Training: A Pilot Study. Computer Vision—ECCV 2018.

[46] Larsen A.B.L., Sønderby S.K., Larochelle H., Winther O. Autoencoding beyond Pixels Using a Learned Similarity Metric. Proceedings of the ICML’16, 33rd International Conference on International Conference on Machine Learning.

[47] Matthey L., Higgins I., Hassabis D., Lerchner A. (2017). dSprites: Disentanglement Testing Sprites Dataset. https://github.com/deepmind/dsprites-dataset/.

[48] Lecun Y., Bottou L., Bengio Y., Haffner P. (1998). Gradient-based learning applied to document recognition. Proc. IEEE.

[49] Liu Z., Luo P., Wang X., Tang X. Deep Learning Face Attributes in the Wild. Proceedings of the International Conference on Computer Vision (ICCV).

[50] Truex S., Baracaldo N., Anwar A., Steinke T., Ludwig H., Zhang R. A Hybrid Approach to Privacy-Preserving Federated Learning. Proceedings of the AISec’19, 12th ACM Workshop on Artificial Intelligence and Security.

[51] van der Maaten L., Hinton G. (2008). Viualizing data using t-SNE. J. Mach. Learn. Res..

